# Morpho-Functional Assessment of Retinal Ganglion Cells and Visual Pathways in Patients with Optic Disc Drusen: Superficial Drusen Visible Height as a Marker of Impairment

**DOI:** 10.3390/jcm12103432

**Published:** 2023-05-12

**Authors:** Giulio Antonelli, Lucia Ziccardi, Lucilla Barbano, Antonio Di Renzo, Vincenzo Parisi

**Affiliations:** IRCCS—Fondazione Bietti, 00198 Rome, Italy; giulio.antonelli@fondazionebietti.it (G.A.); lucilla.barbano@fondazionebietti.it (L.B.); vincenzo.parisi@fondazionebietti.it (V.P.)

**Keywords:** optic disc drusen, PERG, VEP, OCT, RGCs

## Abstract

The aim of this study was to assess the morpho-functional involvement of the retinal ganglion cells (RGCs) and of the visual pathways in patients with superficial (ODD-S) or deep (ODD-D) optic disc drusen. This study enrolled 17 patients with ODD (mean age of 59.10 ± 12.68 years) providing 19 eyes and 20 control subjects (mean age 58.62 ± 8.77 years) providing 20 eyes. We evaluated the following: best-corrected visual acuity, visual field mean deviation (MD), the amplitude (A) of Pattern Electroretinogram (PERG), the implicit time (IT) and A of Visual Evoked Potentials (VEPs), retinal nerve fiber layer thickness (RNFL-T) and ganglion cell thickness (GC-T). In ODD-S eyes, the drusen visible height was measured. ODD-D and ODD-S were detected in 26.3% and 73.7% of ODD eyes, respectively. Significantly (*p* < 0.01) reduced MD, PERG A, VEP amplitude, RNFL-T and GC-T values and significantly (*p* < 0.01) increased VEP IT values were found in the ODD Group as compared to the Control one. In the ODD Group, no significant correlation (*p* > 0.01) between PERG As and VEP ITs was found. In ODD-S, the visible height was significantly correlated (*p* < 0.01) with reduced MD, PERG As and RNFL-T and with increased PSD and VEP IT values. Our findings suggest that ODD might induce morpho-functional changes in RGCs and their fibers and an unrelated visual pathway dysfunction leading or not leading to visual field defects. The observed morpho-functional impairment should be ascribed to an alteration in retrograde (from the axons to the RGCs) and anterograde (from the RGCs up to the visual cortex) axoplasmic transport. In ODD-S eyes, a minimum visible height of 300 microns represented the threshold for the abnormalities, suggesting that “the higher the ODD, the worse the impairment”.

## 1. Introduction

Optic disc drusen (ODD) are congenital acellular deposits of calcium, amino and nucleic acids, and mucopolysaccharides located in front of the lamina cribrosa of the optic nerve [1], with an unknown pathogenesis. The predictive factors of these formations are a narrow scleral canal and a genetic predisposition contributing to axoplasmic metabolism disruption, an extrusion of mitochondria into the extracellular space and an abnormal calcium metabolism [2]. ODD are mainly bilateral, with a female predilection (61–71% of cases) and a prevalence that ranges from 0.4 to 3.7% of the overall population; however, the prevalence of subclinical ODD may be even higher [3,4]. ODD have been associated with several diseases, including retinitis pigmentosa, papilledema, rare types of uveitis, syndromic retinal dystrophies, retinal myelination, benign hamartoma and angioid streaks [4]. Moreover, ODD have been associated with glaucoma, hiding the optic disc cupping typical of glaucoma, leading to a misdiagnosis or a delayed diagnosis [5].

According to the ODD Studies Consortium, ODD can be divided into superficial (ODD-S) and deep (ODD-D), depending on whether the localization is above or below the level of Bruch’s membrane opening (BMO) [6]. ODD-D are not easily detectable in a classical routine ophthalmological examination [6]. By contrast, ODD-S, which are thought to represent 40% of cases [7], can be detected by using autofluorescence due to the presence of calcium and by using ocular ultrasound. With the advent of spectral-domain optical coherence tomography (Sd-OCT) and of enhanced depth imaging OCT (EDI-OCT) in clinical practice, the detection rate of ODD has improved for the highest sensitivity near the inner sclera and the excellent visualization of ODD-D [8]. Even if many patients with ODD do not declare visual symptoms, visual field (VF) defects (arcuate defects, enlargement of the blind spot and concentric contraction) [9] are more commonly described in ODD-S than in ODD-D [10], and a progression of the deficit is possible [10]. 

The impact of ODD on the neural conduction along the visual pathways was studied by using Visual Evoked Potential (VEP) recordings [11,12], often yielding contrasting results, since an increased VEP P100 implicit time was found, ranging from 0% to 83% of ODD cases across studies [13]. Although it is possible to selectively evaluate the neural conduction along small and large axons of the visual pathways by using different visual stimuli [11,12], previous VEP studies have not provided specific information on the impairment of different axons in ODD. 

Moreover, the function of the innermost retinal layers (retinal ganglion cells—RGCs—and their fibers), in a cohort of 24 ODD eyes [14], was assessed using Pattern Electroretinogram (PERG) [15], and the findings showed a reduced N95 wave amplitude in almost 79% of ODD eyes. 

Regarding the impact of ODD on the thickness of the peripapillary retinal fiber nerve layer (RNFL) [7,16,17,18], more recent reports uniquely identified a reduction in RNFL thickness [5,10,19].

Regarding the potential correlations between the volume and size of ODD and the morphologic and functional data of visual pathways [20,21,22,23], RNFL thinning has been found to be significantly correlated with ODD diameter and number [5], and an association between ODD size and VF defects has also been described [22,24].

Nevertheless, there is a lack of comprehensive data on the effects of ODD on the psychophysics and morpho-function of the inner retina and visual pathways. Therefore, the aim of our study was to evaluate whether the occurrence of ODD may induce changes in visual acuity and/or VF defects, morphological and functional impairments in RGCs and their fibers, and/or a dysfunction of the small and large axons of the visual pathways. In addition, in eyes with ODD-S, it was investigated whether the ODD visible height influences the morpho-functional retinal and visual pathway conditions. 

## 2. Materials and Methods

### 2.1. Study Design and Participants

In this retrospective case series, based on the inclusion criteria (see below), we included, between June 2021 and October 2022, seventeen patients (mean age of 59.10 ± 12.68 years, range: 42–78 years) with a clear diagnosis of ODD, made via an ophthalmological evaluation and by using autofluorescence/Ss-OCT imaging and/or ultrasonography. Diagnoses were made at our Clinical and Research Center of Neurophthalmology, Genetic and Rare Diseases of IRCCS—Fondazione Bietti, and the ODD Group was selected based on the inclusion criteria.

We studied 19 ODD eyes from 17 patients, showing bilateral ODD in only 2 cases of ODD-S. 

Furthermore, 20 normal age-similar subjects (mean age of 58.62 ± 8.77 years, range: 40–78 years), providing 20 eyes, served as Controls.

An extensive ophthalmological examination was performed in all Controls and patients with ODD, and it involved an assessment of best-corrected visual acuity (BCVA), an examination of the anterior segment using slit-lamp biomicroscopy, a measurement of intraocular pressure (IOP) and an evaluation of the optic nerve head using indirect ophthalmoscopy and 30° color photography. In addition, the Controls and patients were tested using the Humphrey 30-2 automated VF test (Humphrey Field Analyzer (HFA) 740; Zeiss, San Leandro, CA, USA), swept-source optical coherence tomography (Ss-OCT) and autofluorescence.

The Controls had an IOP of less than 18 mmHg; a BCVA of 0.0 logMAR with a refractive error between −2.00 and +2.00 spherical equivalent; a 30-2 threshold VF with a mean deviation (MD) > −2.0 dB and pattern standard deviation (PSD) < 2.0 dB [25]; and no evidence of an optic disc (or retinal diseases) in the indirect ophthalmoscopy, color photography, Ss-OCT or B-scan ultrasonography.

The inclusion criteria for the patients with ODD were as follows:

(1) Age ranging from 18 to 80 years;
(2) HFA 30-2 VF with defects that preserved the ability to maintain stable fixation comparable to that of normal subjects (fixation loss rate higher than 4%);
(3) Capacity to clearly distinguish a target of fixation placed in the center of the screen, at a viewing distance of 114 cm, in which the visual stimuli of PERG and VEP were presented (see below);
(4) BCVA between 0.00 and 0.40 logarithm of the minimum angle of resolution (logMAR);
(5) Refractive error (when present) between −3.00 and +3.00 spherical equivalent;
(6) IOP less than 18 mmHg;
(7) Absence of cornea, lens, and retina/macula diseases or detectable spontaneous eye movements (i.e., nystagmus).

We excluded from the present study all eyes showing any sign of optic nerve pathology other than ODD. We also excluded eyes with HFA 30-2 defects consisting of a cecocentral scotoma and or central defects enclosing the physiological blind spot. 

In the present retrospective case series study, all procedures adhered to the tenets of the Declaration of Helsinki. The study protocol (NEU_03-2021; 125/21/FB) was approved by the local ethical committee (Comitato Etico Centrale IRCCS Lazio, Sezione IFO/Fondazione Bietti, Rome, Italy), and, upon recruitment, informed consent was obtained from each subject enrolled in the study.

### 2.2. Procedures

#### 2.2.1. Visual Acuity Assessment

Modified Early Treatment Diabetic Retinopathy Study (ETDRS) Tables (Lighthouse, Low vision products, Long Island City, NY, USA) were used to evaluate BCVA at a distance of 4 m. VA is quantified in LogMAR values. 

#### 2.2.2. Visual Field Examination

Static perimetry was performed by using the HFA 30-2 SITA Standard strategy, and MD (in dB) and PSD (in dB) values were obtained. Exams showing a fixation loss rate higher than 4% were discarded.

#### 2.2.3. Electrophysiological Examinations 

According to our previously published studies [26,27,28,29,30,31], simultaneous PERGs and VEPs were carried out by using our previously published methods. 

Briefly, visual stimuli were presented monocularly on a TV monitor subtending 23 degrees at a distance of 114 cm, and they consisted of a checkerboard pattern with a contrast of 80% and mean luminance of 110 cd/m^2^. In the center of the monitor, a small fixation target was placed. The check edges subtended 60 min (60′) and 15 min (15′) of the visual angle and were reversed in contrast at 2 reversals per second. All electrophysiological procedures followed the International Society for Clinical Electrophysiology of Vision (ISCEV)’s standards [11]. We used two different checkerboard patterns to obtain a predominant activation of larger (60′ checks) or smaller (15′ checks) axons [26,27,29,32].

##### PERG Recordings

PERG responses were recorded by using two skin Ag/AgCl electrodes placed over the lower eyelid, one as an active electrode on the open stimulated eye and one as a reference electrode on the pathed eye [33]. The ground electrode was in Fpz [34]. The interelectrode resistance was lower than 3000 ohms. The signal was amplified (gain of 50,000), filtered (band pass of 1–30Hz) and averaged with the automatic rejection of artefacts (100 events free from artefacts were averaged for every trial) using CSO (CSO, Firenze, Italy). The analysis time was 250 msec. In the analysis of the PERG responses, we considered the peak-to-peak amplitude between the first positive peak (P50) and the second negative peak (N95). The PERG P50-N95 amplitude (PERG A) was measured in microvolt.

##### VEP Recordings

VEP responses were recorded by using Ag/AgCl electrodes placed in Oz (active electrode) and in Fpz (reference electrode) [34], and the ground electrode was placed on the left arm. The interelectrode resistance was kept below 3000 ohms. The bioelectric signal was amplified (gain of 20,000), filtered (band pass of 1–100 Hz) and averaged (200 events free from artefacts were averaged for every trial) using CSO. The analysis time was 250 ms. In the analysis of the VEP responses, we considered the implicit time (VEP IT) of the first positive peak (P100) = mea and the peak-to-peak amplitude between the first negative peak (N75) and the P100 peak. The VEP N75-P100 amplitude (VEP A) was measured in microvolt. 

For the PERG and VEP responses, we measured the signal-to-noise ratio (SNR) following our previous published works [26,27,28,29,30,31]. In all subjects and patients, we accepted PERG and VEP signals with a signal-to-noise ratio >2.

#### 2.2.4. Spectral-Domain Optical Coherence Tomography 

The peripapillary RNFL thickness and the structural condition of the RGCs were investigated by performing swept-source optical coherence tomography (Ss-OCT) (Topcon DRI OCT Triton, Topcon, Japan) after pupil dilation with tropicamide 1% eye drops. Each scan was carefully reviewed for the accurate identification and segmentation of the retinal layers by two expert graders (L.Z. and L.B.). We followed the APOSTEL recommendations for quality control [35]. The acceptable signal strength index of the acquired scan was at least 40. The macular ganglion cell complex (GCC) was segmented by using the macula 3D scan (V) protocol with the following parameters: an area of 7.0 × 7.0 mm centered on the fovea and a scan density of 512 (vertical) × 128 (horizontal) scans. Only the GC-Inner Plexiform thickness was taken into consideration (GCL+). Peripapillary RNFL was recorded as superior, inferior, temporal, nasale or overall components using a 6.0 × 6.0 mm area centered on the optic nerve with a scan density of 512 (vertical) × 128 (horizontal) scans. In the Ss-OCT results, we assessed the averaged values of RNFL thickness (RNFL-T measured in microns) from superior (RNFL-ST), inferior (RNFL-IT), nasal (RNFL-NT) and temporal (RNFL-TT) quadrants, and the data obtained from the average of all quadrants (average of 4 values) were identified as RNFL overall (RNFL-OT); the GCC thickness (GC) was also measured. 

According to The Optic Disc Drusen Studies Consortium [6], we classified ODD as ODD-S or ODD-D by using Ss-OCT. ODD were detected as acellular deposits visualized on B-scan Ss-OCT images. ODD were defined as a mass with a boundary, which contrasted with the normal optic nerve head anatomy; they did not belong to the normal retinal layers above BMO, or they contrasted with the prelaminar tissue beneath BMO. In ODD-S eyes, the ODD visible height was measured as the perpendicular distance of ODD protruding above BMO [21]. With ODD-D being located below BMO, no measurement could be obtained. The B-scan Ss-OCT image with the largest height was used for the measurement.

#### 2.2.5. Statistics

A Gaussian distribution of our data was assumed and assessed using the Kolmogorov–Smirnov test. From a pilot study conducted in 8 ODD eyes from 8 subjects and in 8 eyes from 8 Control subjects, other than those included in the current study (unpublished data), we obtained size estimates. Inter-individual variability, expressed as standard deviation (SD) data, was estimated for 15′ PERG A. For 15′ PERG A, the mean/SD ratio value was higher for the Control Group (mean: 2.50 microvolt; SD: 0.41 microvolt, 16.4% of the mean) than for the ODD Group (mean: 1.85 microvolt; SD: 0.45 microvolt, 24.3% of the mean). 

Assuming the above mean and SD values, a sample size of Controls and patients with ODD was established. A power of 90% (β = 10%) at α = 1% detected a between-group difference of 26% in 15′ PERG A. Thus, a sample size of 14 patients with ODD and 18 Control subjects was obtained.

Based on the t-Student distribution for the PERG, VEP and Ss-OCT parameters, 95% confidence limits (CLs) were obtained from the Control data considering superior CL for VEP IT and inferior CL PERG A, VEP A, RNFL-T and GC (see Table 1). 

MD and PSD were considered abnormal for values greater than −2 dB and +2 dB, respectively.

A one-way analysis of variance (ANOVA) was used to assess the differences in the values of the HFA, PERG, VEP and Ss-OCT parameters observed in the ODD and Control Groups. A comparison of electro-functional (PERG A, VEP IT and VEP A), HFA (MD and PSD) and morphological (ODD visible height (in ODD-S eyes), RNFL-T and GC) data was carried out by performing Pearson’s test.

A *p* value lower than 0.01 was considered significant in all statistical analyses. 

All statistical analyses were performed using MedCalc V.13.0.4.0 (MedCalc, Mariakerke, Belgium).

## 3. Results

### 3.1. Demographic and Clinical Features

**Figure 1:**
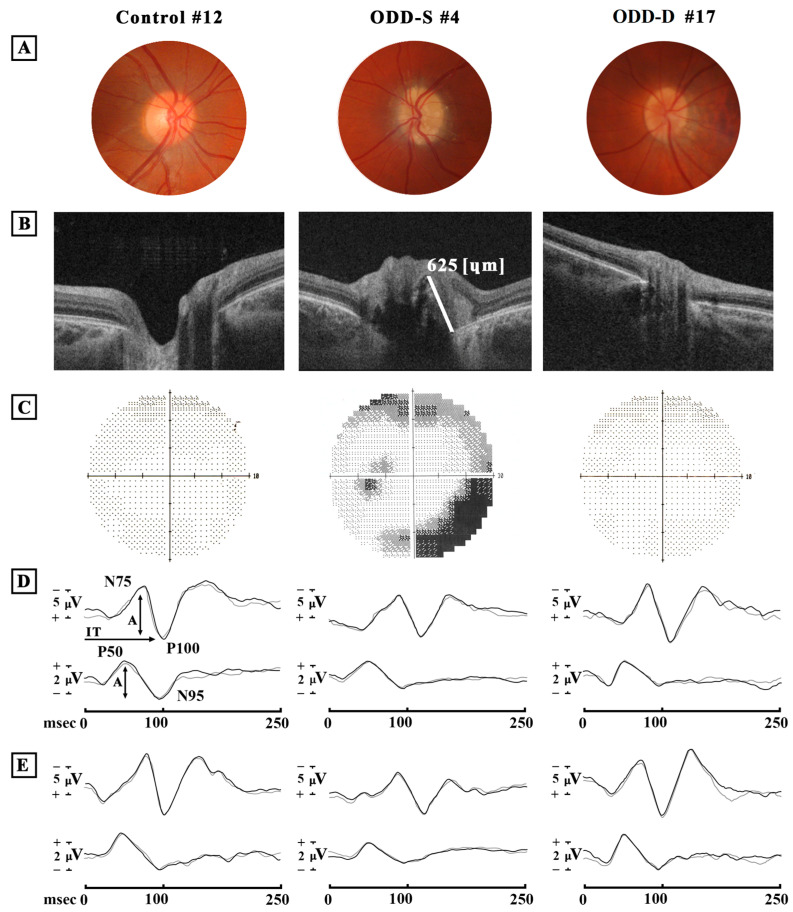
Examples of optic nerve fundus photography (**A**), optical coherence tomography (OCT) B-scan images (**B**), 30-2 Humphrey Field Analyzer (HFA) (**C**), simultaneous recordings of Visual Evoked Potentials (VEPs) and Pattern Electroretinogram (PERG) in response to 60′ (**D**) and 15′ (**E**) visual stimuli checks from one eye of one normal Control subject (Control #12), from one eye with superficial optic disc drusen (ODD-S #4) and from one eye with deep optic disc drusen (ODD-D #17). In ODD-S #4 eye (**B**), the white line indicates the measurement of the ODD visible height (microns). In (**D**,**E**), N75 and P100 refer to the first negative and the first positive peaks of VEP recordings (the implicit time of P100 (→) and the peak-to-peak N75-P100 amplitude (↕) were considered); P50 and N95 refer to the first positive and the second negative peaks of PERG recordings (the peak-to-peak P50-N95 amplitude (↕) was considered). msec = milliseconds; µV = microvolt. When compared to Control eyes, in ODD-S eye, abnormal HFA, reduced 60′ N75-P100 VEP and 60′ and 15′ P50-N95 PERG amplitudes, and delayed 60′ and 15′ P100 VEP implicit times can be observed. By contrast, in ODD-D eye, with respect to Control eye, similar HFA but reduced 60′ P50-N95 PERG amplitude can be found.

Figure 1 presents examples of the VF, PERG and VEP recordings and Ss-OCT scans detected in one representative Control eye (#7) and in one eye with ODD-S (#4).

In Table 1, individual ODD data items (ODD-D and ODD-S with relative visible height), consisting of the HFA, PERG and VEP and Ss-OCT parameters from all 19 eyes, are presented. 

The number and the relative percentage of abnormal values for each psychophysical (HFA 30-2), electrophysiological (PERG and VEP) and morphological (RNFL-T and GC-T) parameter are reported in Table 2. The mean HFA, PERG, VEP, RNFL and GC-T values observed in the Control and ODD Groups and the relative statistical analyses between the Groups are shown in Table 2.

### 3.2. Optic Disc Druse Characteristics 

In our enrolled patients with ODD, we considered ODD-D as cases where the prevalent druse deposits visible in SS-OCT were below BMO and ODD-S as cases where the prevalent druse deposits visible in SS-OCT were above BMO. ODD-D were detected in 5/19 (26.3%) eyes, whereas we found ODD-S in 14/19 (73.7%) eyes. The visible height of ODD-S ranged from 121 to 635 µ (mean of 399.21 ± 142.40 µ). Considering selectively ODD-S eyes, the visible height was not significantly correlated with patient age (r = −0.099, *p* = 0.7362).

### 3.3. Best-Corrected Visual Acuity Data

In all enrolled ODD eyes, we observed a BCVA of 0.0 LogMAR.

### 3.4. Visual Field Changes: HFA Data

None of our patients presented a central scotoma, and, therefore, all of them had a preserved BCVA. Only 7 ODD eyes (36.8%) showed VF defects, whereas in the others (12 eyes, 63.2%), a normal visual field was assessed. The most frequent defects in the ODD eyes with VF defects were reduced peripheral sensitivity (15.8%), peripheral constriction (15.8%) and blind spot enlargement (5.2%). 

Reduced values of MD (<−2 dB) and increased values of PSD (>2 dB) were detected in 7/19 (36.84%) and in 11 (57.89%) ODD eyes, respectively. Reduced MD values were observed in 6/19 (31.58%) ODD-S eyes and in 1 (5.26%) ODD-D eye, whereas increased PSD values were found in 8/19 (42.11%) ODD-S eyes and in 3 (15.79%) ODD-D eyes. 

**Figure 2:**
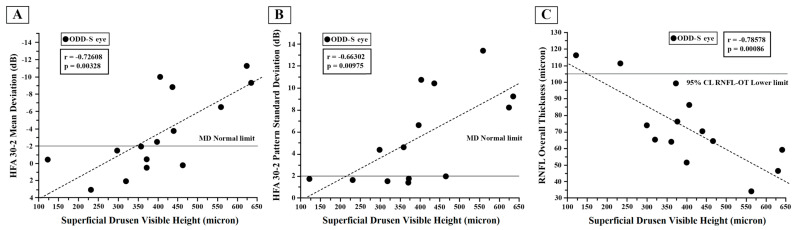
Individual values of superficial optic disc drusen (ODD-S) visible height plotted as a function of the corresponding values of Humphrey 30-2 automated visual field test (HFA) mean deviation (**A**), of pattern standard deviation (**B**) and of retinal nerve fiber layer (RNFL) Overall Thickness (**C**). The solid line indicates the normal limit in (**A**,B) and the 95% lower normal limit in (**C**). The dashed line indicates the regression analysis and correlations for which Pearson’s test was used.

On average, significantly (*p* < 0.01) reduced MD and increased PSD mean values were found in the ODD Group with respect to those found in the Control Group. As shown in Figure 2A,B, in the ODD-S eyes, the reduction in MD and the increase in PSD were significantly (*p* < 0.01) correlated with the ODD visible height. 

### 3.5. Retinal Ganglion Cells’ Functional Changes: PERG Data

Reduced values of 60′ and 15′ PERG As were observed in 14/19 (73.68%) and in 16/19 (84.21%) ODD eyes, respectively. The 60′ PERG A was reduced in 11 (57.89%) ODD-S eyes and in 3 (15.79%) ODD-D eyes; similarly, 15′ PERG A was abnormal in 13 (68.42%) ODD-S eyes and in 3 (15.79%) ODD-D eyes. On average, significantly (*p* < 0.01) reduced mean values of 60′ and 15′ PERG As were detected in the ODD Group with respect to those detected in the Control Group. 

**Figure 3:**
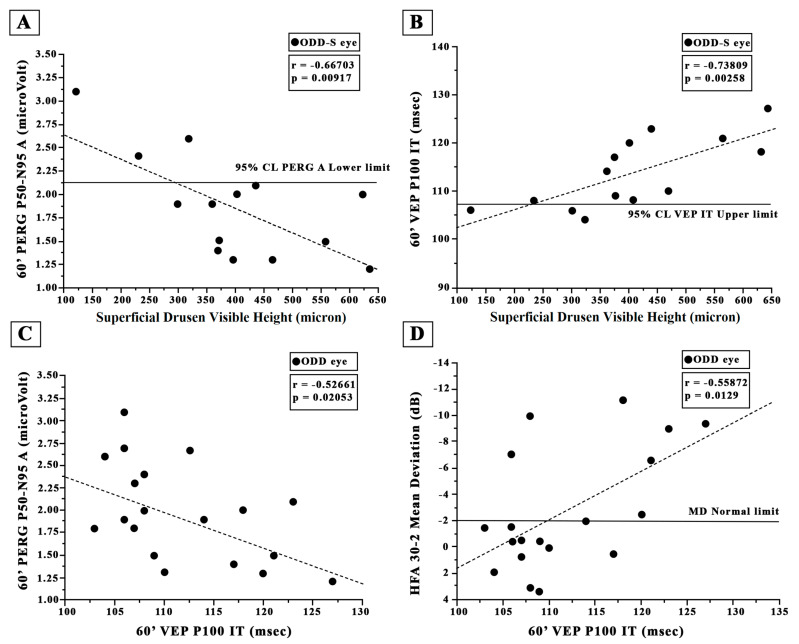
Individual values of superficial optic disc drusen (ODD-S) visible height plotted as a function of (**A**) the corresponding values of 60′ Pattern Electroretinogram (PERG) P50-N95 amplitude (**A**,**B**) and of 60′ Visual Evoked Potential (VEP) P100 implicit time (IT). The solid line indicates the 95% lower normal limit in (**A**) and the 95% upper normal limit in (**B**). Individual values of 60′ Visual Evoked Potential (VEP) P100 implicit time (IT) detected in all eyes with optic disc drusen (ODD) plotted as a function of the corresponding values of 60′ PERG P50-N95 A (**C**) and Humphrey 30-2 automated visual field test (HFA) mean deviation (**D**). The dashed line indicates the regression analysis and the correlations obtained with Pearson’s test. 60′ refers to the visual stimuli checks subtending 60 min of visual arc.

As shown in Figure 2A and Figure 3A, in the ODD-S eyes, both 60′ and 15′ reduced PERG As were significantly (*p* < 0.01) linearly correlated with the increase in the ODD visible height. It is worth noting that a greater percentage of reduced PERG A was detected in eyes with an ODD visible height greater than 300 microns. 

### 3.6. Neural Conduction along the Visual Pathways’ Changes: VEP Data

An increase in the 60′ and 15′ VEP IT values were found in 12 (63.16%) and in 10 (52.63%) ODD eyes, respectively. We found that 60′ VEP ITs were abnormal in 11 (57.89%) ODD-S eyes and only in 1 (5.26%) ODD-D eye; similarly, 15′ VEP ITs were found to be abnormal in 8 (42.11%) ODD-S eyes and in 2 (10.53%) ODD-D eyes. On average, the mean values of 60′ and 15′ VEP ITs were significantly (*p* < 0.01) increased in the ODD Group compared with those in the Control Group. Reduced VEP As were recorded in 11 (57.89%) ODD eyes for both 60′ and 15′ stimuli. Abnormal values were recorded in 8 (42.11%) ODD-S eyes and in 3 (15.79%) ODD-D eyes for 60′ VEP, whereas for 15′ VEP A values, 9 (47.37%) ODD-S and 3 (15.79%) ODD-D eyes were found to be abnormal. On average, the 60′ and 15′ VEP A mean values of the ODD Group were significantly (*p* < 0.01) reduced when compared to those of the Control Group. 

**Figure 4:**
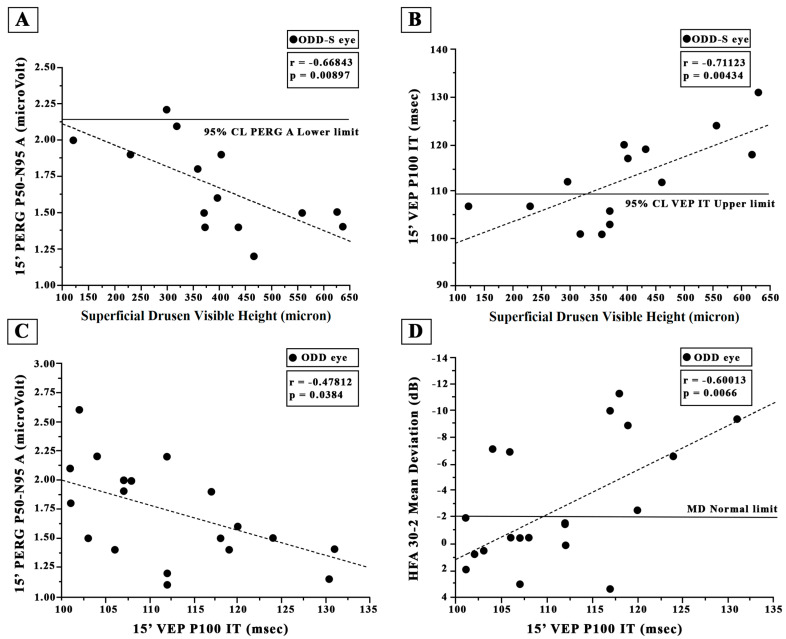
Individual values of superficial optic disc drusen (ODD-S) visible height plotted as a function of (**A**) the corresponding values of 15′ Pattern Electroretinogram (PERG) P50-N95 amplitude (**A**,**B**) and of 15′ Visual Evoked Potential (VEP) P100 implicit time (IT). The solid line indicates the 95% lower normal limit in (**A**) and the 95% upper normal limit in (**B**). Individual values of 15′ Visual Evoked Potential (VEP) P100 implicit time (IT) detected in all eyes with optic disc drusen (ODD) plotted as a function of the corresponding values of 15′ PERG P50-N95 A (**C**) and Humphrey 30-2 automated visual field test (HFA) mean deviation (**D**). The dashed line indicates the regression analysis and the correlations obtained with Pearson’s test. 15′ refers to the visual stimuli checks subtending 15 min of visual arc.

In the ODD-S eyes, as presented in Figure 3B and Figure 4B, both 60′ and 15′ VEP ITs were significantly (*p* < 0.01) linearly correlated with the ODD visible height, and it is worth noting that a greater percentage of increased VEP ITs was detected in ODD eyes with a visible height greater than 300 microns.

Considering all 19 ODD eyes, the 60′ and 15′ VEP ITs values were not significantly (*p* > 0.01) correlated with the 60′ and 15′ PERG A values (see Figure 3C and Figure 4C). As shown in Figure 3D and Figure 4D, the 60′ VEP IT values were associated but not significantly correlated (*p* > 0.01) with the MD values, whereas a significant (*p* > 0.01) correlation between 15′ VEP ITs and the MD values was found; both the 60′ and 15′ VEP IT values were significantly correlated with the PSD values (r = −0.645, r = 0.002 and r = 0.6854, *p* = 0.0012).

### 3.7. Retinal Nerve Fiber Layer and Ganglion Cell Thicknesses: OCT Data

A reduced RNFL thickness was found in the ODD eyes: RNFL-TT was reduced in 16 eyes (84.21%), RNFL-ST was reduced in 14 eyes (73.68%), RNFL-IT was reduced in all 19 eyes (100%), RNFL-NT was reduced in 5 eyes (26.31%), and RNFL-OT was reduced in 16 eyes (94.74%). Regarding the involvement of each sector, abnormal thickness values were found in 9 (47.37%) ODD-S eyes and in 3 (15.79%) ODD-D eyes for RNFL-TT; in 11 (67.89%) ODD-S eyes and in 3 (15.79%) ODD-D eyes for RNFL-ST; in 14 (73.68%) ODD-S eyes and 5 (26.32%) ODD-D eyes for RNFL-IT; in 5 (26.32%) ODD-S eyes and in 1 (5.26%) ODD-D eye for RNFL-NT; and in 12 (63.16%) ODD-S eyes and in 4 (21.05%) ODD-D eyes for RNFL-OT. GC-T was found to be reduced in 13 ODD eyes (73.68%), more specifically, in 10 (52.63%) ODD-S and in 3 (15.79%) ODD-D eyes, respectively. On average, in the ODD Group, the mean RNFL thickness values were significantly reduced (*p* < 0.01) as compared to those of the Controls in several sectors (RNFL-TT, ST, OT and IT) apart from the nasal one; for instance, RNFL-NT was not statistically different (*p* = 0.986) when compared to that in the Controls. 

Considering selectively ODD-S eyes, the visible height was linearly significantly correlated with the RNFL-OT (*p* < 0.01, see Figure 2C) and RNFL-NT (r = −0.815, *p* = 0.0003) values. The ODD visible height was not significantly correlated with the RNFL-TT (r = −0.477, *p* = 0.0873), RNFL-ST (r = −0.648, *p* = 0.0120), RNFL-IT (r = −0.558, *p* = 0.0377) or GC-T (r = −0.407, *p* = 0.1482) values.

## 4. Discussion

To provide a contribution for a better understanding of the impact of ODD on the morpho-functional condition of different elements forming the visual pathways, our aim was to assess whether the presence of ODD (superficial or deep) could induce changes in BCVA and/or VF defects, morphological and functional impairments in RGCs and their fibers, and/or a dysfunction of the neural conduction along the small and large axons of the visual pathways. Nevertheless, due to the small number of observed ODD-D (n = 5) cases, not sufficient to provide an adequate sample size (see the Statistics Section), we were not able to make a statistical comparison between the ODD-S and ODD-D subgroups. In the eyes with ODD-S, the relevance of the visible height to the psychophysical, electrophysiological and morphological findings was also investigated.

Briefly, in the ODD Group, as compared to the Controls, we observed the following major findings:

(1) A normal BCVA (0.0 LogMAR) but a significantly reduced HFA MD and increased HFA PSD values, correlated with increased 15′ VEP ITs;
(2) Significantly reduced 60′ and 15′ PERG and VEP A values and significantly increased 60′ and 15′ VEP IT values;
(3) A significantly reduced RNFL-T in all sectors apart from the nasal one and a reduced GC-T.

In the ODD-S eyes, the values of perimetric changes, the reduced PERG As, the increased VEP ITs and the reduction in RNFL-OT were significantly linearly correlated with the ODD visible height. VF, PERG, VEP and RNFL-OT changes were greater for an ODD visible height > 300 microns. Surprisingly, most of our patients presented with unilateral drusen and not bilateral drusen, as expected. It is likely that Ss-OCT was not as able as EDI-OCT to detect small and deep drusen; therefore, it is possible that small or deep drusen of the contralateral eye were not detected.

### 4.1. ODD and Visual Field Changes: HFA Data 

Even though our patients with ODD did not report any visual disturbances, VF defects were found in almost one-third (36.8%) of the studied ODD eyes (regarding the HFA MD values), and perimetric changes were mainly identified in the ODD-S eyes. 

This agrees with all that reported in the literature, since ODD is almost always an incidental finding during an ophthalmological examination. VF defects have been associated with ODD in 51% of children [13], 75% of affected individuals overall [36] and up to 87% of cases [37], being more commonly observed in ODD-S eyes [36,38,39,40,41], with a trend of progression during adolescence and minimal worsening thereafter [42,43]. VF defects, most commonly enlarged blind spots, aspecific defects, bundle defects and concentric narrowing [21], have been suggested to be due to the compression of the RGCs’ fibers by calcified ODD, leading to RGC death and axonal degeneration [16], the blockage of axonal transport [2,44] or, alternatively, vascular failure [13]. In any case, ODD make patients more susceptible to vision loss due to anterior ischemic optic neuropathy [37,45], central retinal artery occlusion [46], central retinal vein occlusion [47] and choroidal neo-vascularization [48]. However, ODD-D have been associated with preserved VF in many cases (95% of eyes examined by Katz and Pomeranz) [16].

Moreover, in the ODD-S eyes, the reduction in MD and the increase in the PSD values were significantly (*p* < 0.01) correlated with the ODD visible height, so the higher the ODD, the worse the perimetric impairment. Our results agree with those reported by Malmqvist et al., who suggested that a worse VF defect is associated with a larger volume of visible ODD, applying a model process going from EDI-OCT scans to the 3D volume of ODD [22]. 

Overall, it is worth noting that a large percentage (63.2%) of ODD eyes showed normal HFA MD and PSD values despite abnormal values of the PERG or VEP parameters being detected in the same eyes. This finding is similar to that observed in patients with ocular hypertension (OHT), in whom PERG and VEP abnormalities were detected despite the presence of unaltered HFA sensitivity [30,31]. The finding of a PERG A reduction in patients with OHT was explained by the data showing that a loss of at least 20% of RGCs is required to induce a reduction in retinal sensitivity evaluated using HFA [49], and, therefore, RGC dysfunction may precede the observation of RGC death, leading to HFA defects [30,31].

In addition, we found that the reduced HFA MD values were significantly (*p* < 0.01) correlated with the increased 15′ VEP IT values, thus meaning that the presence of ODD produced proportionate disturbances in both VF and the neural conduction of the small axons of the visual pathways. The correlation between the HFA values and the 60′ VEP IT values was near significance (*p* = 0.0129), and this can suggest that the neural conduction along the large axons might also contribute to the reduced HFA MD. 

### 4.2. ODD and Retinal Ganglion Cell Function: PERG Data

In the ODD eyes, the function of RGCs and their fibers was studied using PERG recordings [15,28,33,50]. In the PERG analysis, we measured the P50-N95 amplitude, since this parameter is considered the most specific for assessing RGCs and their fibers’ function [51,52]. Since it was described that PERG P50 IT might be influenced by the pre-ganglionic elements’ function [27,30,51], this parameter was not taken into consideration. The notion that PERG A is a sensitive tool for monitoring RGC function under the compressive effect of ODD was also recently suggested by Pojda-Wilczek et al. [7].

A PERG A reduction was found in more than 70% of individual studied eyes. On average, with respect to the Control eyes, in the ODD Group, we observed a significant reduction in both 15′ and 60′ PERG A. Our findings are consistent with previously reported data [14] of reduced PERG A in ODD eyes. 

Our detected PERG abnormalities can be ascribed to the RGC involvement secondary to a local disturbance of axoplasmic transport at the optic disc for the mechanical compression of prelaminar RNFL caused by ODD and for some retrograde distension of the RGC body [44]. This is also supported by experimental studies of optic neuropathies, in which it was suggested that abnormalities in retrograde axoplasmic transport alter the integrity of PERG A [53]. Thus, based on these concepts and previous findings [53], the PERG A reduction observed in our study suggests that ODD compression may induce an impaired function of the RGCs’ fibers. 

An interesting finding provided by our results was the significant correlation between the 15′ PERG A reduction and the increased ODD-S visible height, suggesting that “the greater the visible height, the worse the RGCs function”. This might mean that ODD-S, localized above BMO, could determine direct compression at the level of intraretinal vasculature inducing localized ischemic damage [19] and, consequently, RGC dysfunction. Based on our data, it should be suggested that a minimum visible height of 300 microns is relevant for inducing RGC abnormalities.

### 4.3. ODD and Neural Conduction along the Visual Pathways: VEP Data

In our ODD eyes, the neural conduction along the large and small axons forming the visual pathways up to the visual cortex was assessed by recording VEP in response to 60′ and 15′ checks [26,27,29]. 

Abnormal values were detected for 60′ and 15′ VEP IT in a large percentage (63.15% and 52.63%, respectively) of individual ODD eyes. A similar condition was found when recording 60′ and 15′ VEP A. On average, with respect to the Control eyes, in the ODD Group, a significant increase in VEP ITs and a significant reduction in VEP A were found. 

Our findings suggest an impairment of the neural conduction along the visual pathways, but this dysfunction was not univocally observed in previous studies, since abnormal VEP IT responses were reported in a wide range (from 0 to 83%) of studied ODD eyes [13,54,55,56].

Our detected VEP abnormalities, according to the study by Malmqivist [22], should be ascribed to hypothesized damage due to mechanical compression on the optic nerve fibers with the consequent involvement of anterograde axonal transport up to the visual cortex. 

In our ODD eyes, we found a lack of correlation between the PERG A and VEP IT responses, thus suggesting that retrograde (impairment of RGCs) and anterograde (abnormal neural conduction along the small and large axons of the visual pathways) axoplasmic transport impairment might occur, but in an unrelated way. 

In the ODD-S eyes, similarly to the HFA and PERG findings, increased 60′ and 15′ VEP ITs were significantly (*p* < 0.01) correlated with the ODD visible height. It is likely that, also in this case, “the higher the ODD, the worse the neural conduction along small and large axons of the visual pathways”, with a relevant effect for ODD visible height values > 300 microns. 

### 4.4. ODD and Retinal Nerve Fiber Layer and Ganglion Cell Thicknesses: OCT Data

With respect to the Control eyes, in the ODD Group, the RNFL thickness was significantly reduced in all sectors but the nasal one. These findings are in agreement with those in a previous report describing an RNFL-T reduction predominantly in other sectors [19], sparing the nasal one, but they are in contrast with those in some other reviews describing a predominance of ODD on the nasal side of the optic disc, with RNFL thinning in the same sector [4,10]. 

RNFL thinning that is associated with the number [5] or the ODD diameter [23] might indicate a process of indirect optic nerve axonal degeneration that can be due to either mechanical compression by the ODD [10] or vascular failure, as suggested by the observed reduced vessel density studied using Ss-OCT angiography in ODD eyes [19]. 

In the absence of a comparison in this study between the ODD-S and ODD-D subgroups, we were not able to compare our findings to those in previous reports finding a greater reduction in RNFL-OT in ODD-S eyes than in ODD-D eyes [17,38,39]. 

In our ODD-S eyes, as for the HFA, PERG and VEP findings, a linear significant correlation between the ODD visible height and the reduction in RNFL-OT was detected, suggesting that “the higher the ODD, the worse the RNFL integrity”. Also in this case, a visible height > 300 microns might be suggested as relevant to inducing a structural impairment.

Regarding the reduced GC-T observed in our ODD Group when compared to the Control one, this finding is in agreement with that in other previous works [4,19,57]. We suppose that this morphological change may be due to all mechanisms previously suggested to explain the PERG A reduction, such as those briefly summarized as retrograde neurodegeneration after compression.

Nevertheless, since PERG represents an electrophysiological tool for the functional assessment of RGCs located in the entire retina and the CG thickness was measured from the macular area, we believed that it was not appropriate to perform a correlation between the functional (PERG A) and morphological (GC-T) findings.

Moreover, in the ODD-S eyes, unlike the RNFL thickness, the GC-T reduction was not significantly correlated with the ODD visible height.

It has been suggested that a macular GC analysis might be more useful for detecting early structural involvement with respect to RNFL thickness analyses, particularly in ODD-D eyes, because ODD-D often produce an RNFL thickening that can mask axonal damage, yielding a false negative result [57]. Regarding this, however, our study cannot provide results confirming this suggested evidence.

## 5. Conclusions

In conclusion, our findings suggest that the presence of ODD might induce morphological changes in RGCs and their fibers (as for the reduced RFNFL and GC-T) and a dysfunction of RGCs and their fibers (as derived by abnormal PERG responses) and an unrelated impairment of neural conduction along the large and small axons forming the visual pathways (indicated by abnormal VEP responses). All these changes either lead or do not lead to visual field defects. The observed morpho-functional impairment should be ascribed to alterations in retrograde (from the axons to the RGCs) and anterograde (from the RGCs up to the visual cortex) axoplasmic transport. In ODD-S eyes, a visible height of 300 microns represented the threshold for the abnormalities, suggesting that “the higher the ODD, the worse the impairment”.

We acknowledge that this study may present some limitations. These are the reduced number of participants; the absence of a follow-up; the non-standardized method of measurement for the visible height of ODD; and the use of SS-OCT instead of EDI (enhanced depth imaging) OCT, which allows for a better visualization of ODD with less artifacts. Regarding this point, however, a recent study showed no significant difference between SS-OCT and the EDI-OCT acquisitions in detecting ODD [58].

## Figures and Tables

**Table 1:** Individual data of optic disc drusen characteristics (type and visible height), 30-2 Humphrey Field Analyzer mean deviation and pattern standard deviation, 60′ and 15′ Pattern Electroretinogram P50-N95 amplitude, Visual Evoked Potential P100 implicit time and N75-P100 amplitude, and sectorial retinal nerve fiber layer thickness and ganglion cell thickness detected in all 19 studied eyes with optic disc drusen.

	**ODD ^a^**		**HFA ^b^ 30-2**		**60′** **PERG ^c^**	**60′** **VEP ^d^**		**15′** **PERG ^c^**	**15′** **VEP ^d^**		**RNFL ^e^**					**GC-T ^f^**
	**Type**	**Visible Height** **(µ ^g^)**	**MD ^h^** **(dB ^i^)**	**PSD ^l^** **(dB ^i^)**	**A ^m^** **(µV ^n^)**	**IT ^o^** **(msec ^p^)**	**A ^m^** **(µV ^n^)**	**A ^m^** **(µV ^n^)**	**IT ^o^** **(msec ^p^)**	**A ^m^** **(µV ^n^)**	**TT ^q^** **(µ ^g^)**	**ST ^r^** **(µ ^g^)**	**IT ^s^** **(µ ^g^)**	**NT ^t^** **(µ ^g^)**	**OT ^u^** **(µ ^g^)**	**(µ ^g^)**
ODD#1	Deep		**−7.06**	**7.5**	2.7	106	**7.1**	2.2	104	**2.4**	**65**	**68**	**51**	87	**67.75**	**50**
ODD#2	Deep		3.46	1.81	**1.5**	**109**	**6.2**	**1.9**	**117**	**3.7**	79	138	**73**	152	110.50	67
ODD#3	Superficial	559	**−6.57**	**13.41**	**1.5**	**121**	**4.2**	**1.5**	**124**	**2.7**	**32**	**44**	**20**	**40**	**34.00**	**54**
ODD#4	Superficial	625	**−11.22**	**8.24**	**2.0**	**118**	**8.1**	**1.5**	**118**	8.1	**33**	**37**	**50**	**65**	**46.25**	56
ODD#5	Superficial	371	0.53	1.45	**1.4**	**117**	**7.5**	**1.5**	103	**4.4**	94	**85**	**68**	150	**99.25**	**50**
ODD#6	Superficial	121	−0.39	1.72	3.1	106	11.5	**2.0**	107	11.6	82	147	**77**	155	116.24	66
ODD#7	Superficial	358	−1.91	**4.62**	**1.9**	**114**	**5.2**	**1.8**	101	**6.3**	**59**	**61**	**51**	84	**63.75**	58
ODD#8	Superficial	397	**−2.43**	**6.62**	**1.3**	**120**	14.8	**1.6**	**120**	15.5	**36**	**56**	**45**	**69**	**51.53**	**50**
ODD#9	Superficial	635	**−9.36**	**9.24**	**1.2**	**127**	**8.2**	**1.4**	**131**	10.7	**72**	**68**	**45**	**52**	**59.25**	**50**
ODD#10	Superficial	464	0.12	1.97	**1.3**	**110**	**5.1**	**1.2**	**112**	**5.5**	82	**67**	**43**	**66**	**64.52**	**55**
ODD#11	Superficial	436	**−8.9**	**10.43**	**2.1**	**123**	**4.2**	**1.4**	**119**	**6.1**	**62**	**84**	**55**	82	**70.75**	**50**
ODD#12	Superficial	404	**−9.97**	**10.74**	**2.0**	**108**	15.3	**1.9**	**117**	**7.2**	**45**	133	**72**	86	**86.28**	**28**
ODD#13	Superficial	372	−0.45	1.72	**1.5**	**109**	9.1	**1.4**	106	**3.5**	81	**63**	**65**	95	**76.05**	**53**
ODD#14	Superficial	298	−1.54	**4.38**	**1.9**	106	**3.2**	2.2	**112**	**4.5**	104	**63**	**30**	100	**74.25**	62
ODD#15	Deep		−0.48	**2.68**	2.3	107	**4.5**	**2**	108	**5.1**	100	**79**	**47**	110	**84.00**	63
ODD#16	Superficial	319	1.96	1.51	2.6	104	16.6	**2.1**	101	12.2	**62**	**56**	**57**	87	**65.50**	**52**
ODD#17	Deep		0.78	**2.37**	**1.8**	107	11.5	2.6	102	10.5	**64**	**87**	**63**	82	**74.00**	**55**
ODD#18	Superficial	230	3.08	1.62	2.4	**108**	18.7	**1.9**	107	10.6	**68**	149	**75**	153	111.25	69
ODD#19	Deep		−1.45	1.38	**1.8**	103	10	**1.1**	**112**	13	**60**	133	**58**	139	**97.50**	**55**
95%CL **^v^**			−2.0	2.0	2.14	107.19	8.38	2.14	109.59	7.48	76.65	109.74	119.13	78.68	104.73	56.00

^a^ ODD = optic disc drusen; ^b^ HFA = Humphrey Field Analyzer, ^c^ PERG = Pattern Electroretinogram, ^d^ VEP = Visual Evoked Potential; ^e^ RNFL = retinal nerve fiber layer; ^f^ GC-T = ganglion cell thickness; ^g^ µ = microns; ^h^ MD = mean deviation, ^i^ dB = decibel; ^l^ PSD = pattern standard deviation 60′; 60′ and 15′ = visual stimuli checks subtending 60 and 15 min of visual arc; ^m^ A = amplitude; ^n^ µV = microvolt; ^o^ IT = implicit time; ^p^ msec = milliseconds; ^q^ TT = Temporal Thickness; ^r^ ST = Superior Thickness; ^s^ IT = Inferior Thickness; ^t^ NT = Nasal Thickness; ^u^ OT = Overall Thickness; ^v^ CL 95%: = confidence limits obtained from Control data considering upper confidence limit for VEP IT and lower confidence limit for PERG A, VEP A, RNFL and GC-T. MD and PSD were considered abnormal for values greater than −2 dB and +2 dB, respectively. The abnormal values with respect to the 95% confidence limits are reported in bold.

**Table 2:** Mean values of age, 30-2 Humphrey Field Analyzer mean deviation and pattern standard deviation, 60′ and 15′ Pattern Electroretinogram P50-N95 amplitude, Visual Evoked Potential P100 implicit time and N75-P100 amplitude, sectorial retinal nerve fiber layer thickness (RNFL), and ganglion cell thickness (GC-T) detected in the Control Group and in the Group of eyes with optic disc drusen.

	Controls (N ^a^ = 20)		ODD ^b^ (N ^a^ = 19)		ODD ^b^ *Ab* ^c^ (N ^a^)	*Ab* ^c^ %	ANOVA ^d^: ODD ^b^ vs. Controls	
	Mean	1SD ^e^	Mean	1SD ^e^			f (1,38) =	*p*=
Age (years)	58.62	8.77	59.10	12.68			0.02	0.894
HFA ^f^ MD ^g^ (dB ^i^)	0.82	0.38	−2.69	4.50	7	36.84	12.09	<0.001
HFA ^f^ PSD ^h^ (dB ^i^)	0.68	1.36	4.77	3.85	11	57.89	19.97	<0.0001
60′ PERG ^l^ A ^m^ (µV ^n^)	2.68	0.27	1.92	0.52	14	73.68	33.30	<0.0001
60′ VEP ^o^ IT ^p^ (msec ^q^)	101.29	2.95	111.51	7.00	12	63.15	35.95	<0.0001
60′ VEP ^o^ A ^m^ (µV ^n^)	13.4	2.51	8.97	4.49	11	57.89	14.66	<0.0001
15′ PERG ^i^ A ^l^ (µV ^n^)	2.66	0.26	1.77	0.39	16	84.21	71.00	<0.0001
15′ VEP ^o^ IT ^p^ (msec ^q^)	104.21	2.69	111.53	8.25	10	52.63	14.18	<0.0001
15′ VEP ^o^ A ^l^ (µV ^n^)	12.52	2.52	7.75	3.80	11	57.89	21.55	<0.0001
RNFL ^r^-TT ^s^ (µ)	86.39	4.87	67.83	20.63	12	63.15	15.31	<0.0001
RNFL ^r^-ST ^t^ (µ)	136.22	13.24	86.39	35.60	14	73.68	34.24	<0.0001
RNFL ^r^-IT ^u^ (µ)	142.37	11.62	58.21	20.60	19	100.00	250.24	<0.0001
RNFL ^r^-NT ^v^ (µ)	97.42	9.37	96.63	35.24	5	26.31	0.01	0.923
RNFL ^r^-OT ^w^ (µ)	116.21	5.74	77.87	22.95	16	84.21	52.43	<0.0001
GC-T ^x^ (µ)	62.4	3.2	54.95	8.74	13	68.42	12.75	0.001

^a^ N = number of eyes, ^b^ ODD = optic disc drusen; ^c^ Ab = abnormal with respect to 95% confidence limits (MD and PSD were considered Ab for values lower or greater + or −2 dB, respectively); ^d^ ANOVA = one-way analysis of variance; ^e^ SD = 1 standard deviation ^f^ HFA = 30-2 Humphrey Field Analyzer, ^g^ MD = mean deviation; ^h^ PSD = pattern standard deviation ^i^ dB = decibel; 60′ and 15′ = visual stimuli checks subtending 60 and 15 min of visual arc; ^l^ PERG = Pattern Electroretinogram; ^m^ A = amplitude; ^n^ µV = microvolt; ^o^ VEP = Visual Evoked Potential; ^p^ IT = implicit time; ^q^ msec = milliseconds; ^r^ RNFL = retinal nerve fiber layer; ^s^ TT = Temporal Thickness; ^t^ ST = Superior Thickness; ^u^ IT = Inferior Thickness; ^v^ NT = Nasal Thickness; ^w^ OT = Overall Thickness. ^x^ GC-T = ganglion cell thickness.

## Data Availability

Available on request.

## Abbreviations

ODD	Optic disc drusen
ODD-S	Superficial optic disc drusen
ODD-D	Deep optic disc drusen
EDI-OCT	Enhanced depth imaging OCT
RGCs	Retinal ganglion cells
PERG	Pattern Electroretinogram
VEP	Visual Evoked Potential
CL	Confidence limit
ANOVA	One-way analysis of variance
RNFL	Retinal nerve fiber layer
BCVA	Best-corrected visual acuity measurement
ETDRS	Early Treatment Diabetic Retinopathy Study
LogMAR	Logarithm of the minimum angle of resolution
IOP	Intraocular pressure
HFA	Humphrey Field Analyzer
MD	Mean deviation
PSD	Pattern standard deviation
ISCEV	International Society for Clinical Electrophysiology of Vision
PERG A	Pattern Electroretinogram amplitude
VEP IT	Visual Evoked Potential implicit time
VEP A	Visual Evoked Potential amplitude
SNR	Signal-to-noise ratio
BMO	Bruch’s membrane opening
SD	Standard deviation
GC	Ganglion cell
OHT	Ocular hypertension
VF	Visual field
SS-OCT	Swept-source optical coherence tomography
TT	Temporal Thickness
ST	Superior Thickness
IT	Inferior Thickness
NT	Nasal Thickness
